# P-1227. Population Pharmacokinetics (PK) of Cefiderocol (FDC) during Acute Pulmonary Exacerbations (APE) in Adult Persons with Cystic Fibrosis (CF)

**DOI:** 10.1093/ofid/ofae631.1409

**Published:** 2025-01-29

**Authors:** Christina Konig, Marguerite Monogue, Ryan K Shields, Colleen M Sakon, Andrew J Fratoni, Hanna Roenfanz, James D Finklea, Samuel Pope, David P Nicolau, Joseph L Kuti

**Affiliations:** Hartford Hospital, Hartford, Connecticut; University of Texas Southwestern Medical Center, Dallas, TX; University of Pittsburgh, Pittsburgh, Pennsylvania; Indiana University Health, Indianapolis, Indiana; Hartford Hospital, Hartford, Connecticut; Hartford Hospital, Hartford, Connecticut; University of Texas Southwestern, Dallas, TX; Division of Pulmonary and Critical Care Medicine, Hartford, Connecticut; Hartford Hospital, Hartford, Connecticut; Hartford Hospital, Hartford, Connecticut

## Abstract

**Background:**

FDC has potent activity against many Gram-negative rods associated with CF APE. Due to metabolic changes seen in CF as well as frequent infection with resistant bacteria, persons with CF (PwCF) frequently have altered PK and may require higher doses. Herein, we describe the population PK and safety of FDC in adult PwCF admitted with an APE.

**Methods:**

Adult PwCF admitted with an APE received at least 3 doses of FDC according to their calculated creatinine clearance (CrCL) by Cockroft-Gault; all doses were infused over 3 hours. Subsequent blood sampling (0, 1.5, 3. 3.25, 3.5, 4, 5, 6,and 8h) was performed after the 3^rd^ - 6^th^ dose. FDC concentrations were analyzed by a validated LC/MSMS method.Protein binding (PB) was determined by ultrafiltration. FDC concentrations were fitted using the non-parametric adaptive grid algorithm in Pmetrics for R. The free time above the MIC (*f*T>MIC) for each PwCF was assessed at MICs of 4, 8, and 16 mg/L. Total AUC_24h_ was calculated to compare overall exposures with non-CF patients. Tolerability and adverse events were monitored throughout the study.

**Results:**

Nine PwCF were included; six received 2g q8h and three 2g q6h based on CrCL. The mean (range) age, weight, and CrCL were 33 (22-58) years, 62 (45-78) kg, and 117 (71-164) mL/min, respectively. A two-compartment model best fit the data (AIC 514) with mean estimates for clearance, volume of the central compartment, and inter compartmental transfer constants (K_12_ and K_21_) of 5.69 ± 1.45 L/h, 7.42 ± 3.74 L, 2.54 ± 1.76 h^-1^ and 2.72 ± 3.04 h^-1^, respectively. PB was 45% (38-51). The 2g q8h regimen for PwCF with CrCL 60-120 mL/min achieved a mean (range) *f*T > MIC of 100% (100-100), 92% (78-100), and 73% (50-100) at MICs of 4, 8, and 16mg/L, respectively, with AUC_24h_ of 1210 (790-1460) mg/L*h. In those with CrCL >120mL/min, the 2g q6h attained 100% *f*T> MIC in all PwCF up to 8 mg/L and 83% (71-92) *f*T > MIC at 16 mg/L, with AUC_24h_ of 1241 (950-1578) mg/L*h. One adverse event of nausea was reported.

**Conclusion:**

Among these 9 PwCF experiencing an APE with normal or augmented renal clearance, FDC using label prescribed dosing regimens according to CrCL was well tolerated and achieved optimal *f*T >MIC exposure in all patients for pathogens up to MICs of 8mg/L. The AUC_24h_ was also similar to previously reported estimates in non-CF patients.

**Disclosures:**

**Christina Konig, PhD**, Gilead Inc.: Honoraria|Pfizer Inc.: Honoraria|Shionogi Inc.: Honoraria **Ryan K. Shields, PharmD, MS**, Allergan: Advisor/Consultant|Cidara: Advisor/Consultant|Entasis: Advisor/Consultant|GSK: Advisor/Consultant|Melinta: Advisor/Consultant|Melinta: Grant/Research Support|Menarini: Advisor/Consultant|Merck: Advisor/Consultant|Merck: Grant/Research Support|Pfizer: Advisor/Consultant|Roche: Grant/Research Support|Shionogi: Advisor/Consultant|Shionogi: Grant/Research Support|Utility: Advisor/Consultant|Venatorx: Advisor/Consultant|Venatorx: Grant/Research Support **Andrew J. Fratoni, PharmD**, InsightRX: Grant/Research Support **James D. Finklea, MD**, Cystic Fibrosis Foundation: Grant/Research Support **David P. Nicolau, PharmD**, CARB-X: Grant/Research Support|Innoviva: Grant/Research Support|Innoviva: Honoraria|Merck: Advisor/Consultant|Merck: Grant/Research Support|Merck: Honoraria|Pfizer: Advisor/Consultant|Pfizer: Grant/Research Support|Pfizer: Honoraria|Shionogi: Advisor/Consultant|Shionogi: Grant/Research Support|Shionogi: Honoraria|Venatorx: Grant/Research Support **Joseph L. Kuti, PharmD**, Abbvie: Advisor/Consultant|bioMerieux: Grant/Research Support|Merck: Grant/Research Support|Pfizer: Grant/Research Support|Shionogi Inc: Advisor/Consultant|Shionogi Inc: Grant/Research Support|Shionogi Inc: Honoraria|Venatorx: Grant/Research Support

